# Development of a CLDN18.2-targeting immuno-PET probe for non-invasive imaging in gastrointestinal tumors

**DOI:** 10.1016/j.jpha.2023.02.011

**Published:** 2023-02-28

**Authors:** Yan Chen, Xingguo Hou, Dapeng Li, Jin Ding, Jiayue Liu, Zilei Wang, Fei Teng, Hongjun Li, Fan Zhang, Yi Gu, Steven Yu, Xueming Qian, Zhi Yang, Hua Zhu

**Affiliations:** a Guizhou University Medicine College, Guiyang, 550025, China; b The Ministry of Education Key Laboratory of Carcinogenesis and Translational Research; NMPA Key Laboratory for Research and Evaluation of Radiopharmaceuticals, Department of Nuclear Medicine, Peking University Cancer Hospital & Institute, Beijing, 100142, China; c Department of Biochemistry and Molecular Biology, School of Basic Medical Sciences, Southwest Medical University, Luzhou, Sichuan, 646000, China; d Suzhou Transcenta Therapeutics Co., Ltd, Suzhou, Jiangsu, 215127, China; e Institute of Biomedical Engineering, Peking University Shenzhen Graduate School, Shenzhen, Guangdong, 518055, China

**Keywords:** Claudin18.2, Gastrointestinal cancers, Zirconium-89, Positron emission tomography, Good Manufacturing Practices

## Abstract

Claudin18.2 (CLDN18.2) is a tight junction protein that is overexpressed in a variety of solid tumors such as gastrointestinal cancer and oesophageal cancer. It has been identified as a promising target and a potential biomarker to diagnose tumor, evaluate efficacy, and determine patient prognosis. TST001 is a recombinant humanized CLDN18.2 antibody that selectively binds to the extracellular loop of human Claudin18.2. In this study, we constructed a solid target radionuclide zirconium-89 (^89^Zr) labled-TST001 to detect the expression of in the human stomach cancer BGC823^CLDN18.2^ cell lines. The [^89^Zr]Zr-desferrioxamine (DFO)-TST001 showed high radiochemical purity (RCP, >99%) and specific activity (24.15 ± 1.34 GBq/μmol), and was stable in 5% human serum albumin, and phosphate buffer saline (>85% RCP at 96 h). The EC_50_ values of TST001 and DFO-TST001 were as high as 0.413 ± 0.055 and 0.361 ± 0.058 nM (*P* > 0.05), respectively. The radiotracer had a significantly higher average standard uptake values in CLDN18.2-positive tumors than in CLDN18.2-negative tumors (1.11 ± 0.02 vs. 0.49 ± 0.03, *P* = 0.0016) 2 days post injection (p.i.). BGC823^CLDN18.2^ mice models showed high tumor/muscle ratios 96 h p.i. with [^89^Zr]Zr-DFO-TST001 was much higher than those of the other imaging groups. Immunohistochemistry results showed that BGC823^CLDN18.2^ tumors were highly positive (+++) for CLDN18.2, while those in the BGC823 group did not express CLDN18.2 (−). The results of ex vivo biodistribution studies showed that there was a higher distribution in the BGC823^CLDN18.2^ tumor bearing mice (2.05 ± 0.16 %ID/g) than BGC823 mice (0.69 ± 0.02 %ID/g) and blocking group (0.72 ± 0.02 %ID/g). A dosimetry estimation study showed that the effective dose of [^89^Zr]Zr-DFO-TST001 was 0.0705 mSv/MBq, which is within the range of acceptable doses for nuclear medicine research. Taken together, these results suggest that Good Manufacturing Practices produced by this immuno-positron emission tomography probe can detect CLDN18.2-overexpressing tumors.

## Highlights

• Development of radiolabeled a GMP grade *anti*-CLDN18.2 antibody PET probe.
• High affinity to CLDN18.2 in vitro and in vivo.
• This tracer can noninvasively report CLDN18.2 expression in different tumors.

## Graphical abstract

**Figure:**
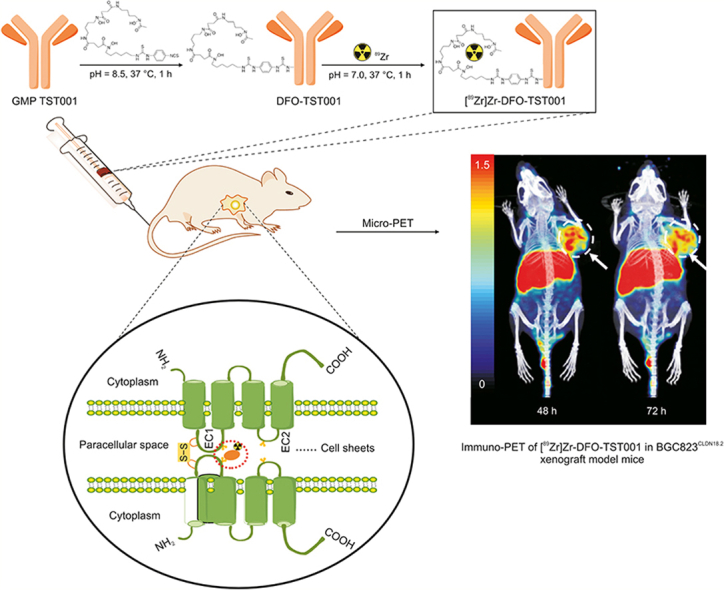

## 1 Introduction

According to the cancer epidemiology report released in 2022, lung cancer is the primary cause of cancer death, followed by digestive tract tumors (such as stomach cancer, colorectal cancer, liver cancer, oesophageal cancer, etc.) [1]. In China, gastrointestinal cancers account for 45% of cancer-related deaths, likely because gastrointestinal cancers are mostly diagnosed in the advanced stage and patients often have a poor prognosis [[2], [3]]. Gastrointestinal cancers have become the primary medical and economic burden for people in China. In addition to traditional chemotherapy and immunotherapy, little progress has been made with novel chemotherapies and targeted therapies for gastrointestinal tumors [[4], [5], [6], [7]]. Among the 70 novel first-line agents approved for cancer treatment, only 5 drugs have been approved for advanced gastrointestinal cancer and the survival rates are still low based on data from the last five years [8]. Therefore, strategies to improve the survival of patients with advanced gastrointestinal cancer remain an unmet medical necessity.

Claudin18.2 (CLDN18.2) is a tight junction protein belonging to the claudin protein family that is involved in the formation of intercellular adhesion structures, and controls cell polarity and the exchange of substances between cells [[9], [10], [11]]. Its expression is strictly limited to normal gastric mucosal cells, but is overexpressed in the process of proliferation, division, and metastasis of tumor cells, making it an emerging therapeutic target for digestive tract tumor therapy [12,13]. Zolbetuximab (IMAB362) is the first targeted CLDN18.2 antibody that kills tumor cells through antibody-dependent cytotoxicity (ADCC) and complement-dependent cytotoxicity, and in combination with first-line epirubicin, oxaliplatin and capecitabine to provide longer progression-free and overall survival [14]. TST001 is an anti-CLDN18.2 monoclonal antibody developed worldwide after IMAB362. Compared to IMAB362, TST001 has a higher affinity and higher FcR binding activity due to lower fucose content and stronger natural killer cell-mediated ADCC tumor killing activity. In a phase I clinical study of TST001 (NCT04396821) in combination with capecitabine and oxaliplatin as a first-line agent for advanced gastric/gastroesophageal junction adenocarcinoma, 73.3% achieved partial response, and 26.7% achieved stable disease [15]. A phase I study (NCT03874897) of CLDN18.2 chimeric antigen receptor-T (CAR-T) therapy conducted by Shen and co-workers [16] showed that after receiving CLDN18.2 CAR-T infusion, the overall response rate and disease control rate reached 48.6% and 73.0%, respectively. Interestingly, both clinical studies indicate that the CLDN18.2 expression level was correlated with drug efficacy, showing more clinical benefit in patients with high CLDN18.2 expression in tumors. Therefore, patient selection based on CLDN18.2 expression level becomes critical for CLDN18.2-targeted therapy. At present, the major detection method of CLDN18.2 protein is immunohistochemistry (IHC), and other methods include molecular beacons and reverse transcription-polymerase chain reaction [17]. IHC is invasive, and requires endoscopic biopsy, and the sampling site and number are limited. Due to the heterogeneous nature of tumor, the CLDN18.2 distribution and dynamic changes in expression levels in patients cannot be fully reflected in real-time. Molecular imaging can be used as a noninvasive diagnostic tool to detect the expression and distribution of CLDN18.2 in the lesion using the radioactive signal emitted by the radiotracer, thereby helping to clinically screen patients with potential benefit, evaluate the efficacy of CLDN18.2 targeted therapy, and guide the accurate diagnosis and treatment of tumors. A recent study showed that ^18^F-fluorodeoxyglucose (FDG) positron emission tomography/computed tomography (PET/CT) parameters including SUVmax, metabolic tumor volume, and total lesion glycolysis did not predict CLDN 18.2 expression status in diffuse-type gastric cancer [18]. Hu et al. [19] developed three antibodies (anti-CLDN18.2 recombinant single-chain antibody fused with poly-histidine-tag (VHH), anti-CLDN18.2 recombinant single-chain antibody fused with albumin binding domain (VHH-ABD), and anti-CLDN18.2 recombinant single-chain antibody fused with IgG1-Fc (VHH-Fc)) of different molecular weight sizes for PET/CT imaging, and identified [^89^Zr]-anti-CLDN18.2 VHH-ABD as the most appropriate imaging agent (high tumor uptake and low uptake in the liver) in preclinical studies. However, in a subsequent clinical study, [^89^Zr]-VHH-Fc was found to be more specific and persistent than [^89^Zr]-anti-CLDN18.2 VHH-ABD, and was also considered to be a molecular imaging tracer with potential value for cancer diagnosis, as it contains CLDN18.2 [20]. More recently, we explored a CLDN18.2-specific murine monoclonal antibody 5C9 by DNA immunization, and modified 5C9 with ^124^I, Cy5.5, and FD1080. The results of these studies support the targeted therapy of CLDN18.2-positive tumors by using immuno-PET imaging and near-infrared fluorescent II imaging to localize tumors and guide surgery for orthotopic CLDN18.2-positive tumors [21].

Due to the superior targeting specificity and high sensitivity of molecular imaging technology, we used the TST001 antibody produced under Good Manufacturing Practices (GMP) conditions to construct the immuno-PET molecular probe [^89^Zr]Zr-desferrioxamine (DFO)-TST001. The goal of this study was to assess the ability of [^89^Zr]Zr-DFO-TST001 to characterize CLDN18.2 expression.

## 2 Material and methods

### 2.1 Materials

All reagents were obtained from Sigma-Aldrich (Shanghai, China). *p*-isothiocyanatobenzyl-desferrioxamine B was purchased from Macrocyclics (Plano, TX, USA). The GMP grade CLDN18.2 antibody TST001 was kindly provided by Suzhou Transcenta Therapeutics Co., Ltd. (Suzhou, China). Radionuclide ^89^Zr was produced and purified by the Cyclotron team of the Nuclear Medicine Department of Peking University Cancer Hospital (Beijing, China). The medium, fetal bovine serum, trypsin ethylene diamine tetraacetic acid, and pen-strep solution were purchased from Biological Industries (Beijing, China). Radioimmunoprecipitation assay lysis buffer was obtained from Themo Fisher Scientific Inc. (Waltham, MA, USA). Diaminobenzidine was provided by Jinqiao Biological Company (Beijing, China). PD-10 column was purchased from GE Healthcare (Buckinghamshire, UK).

### 2.2 Radiolabeling of TST001 with ^89^Zr

For ^89^Zr labeling, ^89^Zr-oxalic acid was neutralized to pH 7.0 using 0.25 M 2-[4-(2-hydroxyethyl)-1-piperazinyl]ethanesulfonic acid and 1 M Na_2_CO_3_ buffer, and then mixed with DFO-TST001 for 60 min at 37 °C. The reaction mixture was purified by a PD-10 column with 0.01 M phosphate buffer saline (PBS, 2.5 mL, pH 7.4).

### 2.3 Small-animal PET imaging of [^89^Zr]Zr-DFO-TST001

Normal Kunming (KM) mice and BGC823^CLDN18.2^/BGC823 model nude mice were injected with 7.4 MBq of [^89^Zr]Zr-DFO-TST001 via the tail vein (*n* = 3). Then 10 min static PET scans were acquired at each time point (2, 24, 48, and 72 h post injection (p.i.)). As a non-specific control group, BGC823^CLDN18.2^ mice (*n* = 3) were fasted 6 h in advance, then injected with 7.4 MBq of ^18^F- FDG via the tail vein. The mice were anesthetized with 2% isoflurane before and during the ^18^F-FDG PET imaging. With a small-animal PET/CT scanner (Super Nova PET/CT, Pingseng Healthcare, Shanghai, China), the PET images were reconstructed by Avatar 3 (Pingseng Healthcare), and the regions of interest (ROIs)-derived SUV was calculated by drawing ROIs over these organs. All animal experiments were performed according to the National Institutes of Health guidelines for the Care and Use of Laboratory Animals and approved by the Animal Care and Ethics Committee of Peking University Cancer Hospital (Approval No.: EAEC 2022-01).

### 2.4 Ex vivo biodistribution

The KM mice were intravenously injected with 0.74 MBq of [^89^Zr]Zr-DFO-TST001 via the tail vein and were then sacrificed at 2, 24, 48, 72 and 144 h p.i. (*n* = 4). The tissues including the blood, heart, liver, spleen, lung, kidneys, stomach, intestines, muscle, bone, and brain were dissected. The radioactivity of the tissues was measured using a γ-counter (PerkinElmer, Waltham, MA, USA). The radioactivity of each organ was calculated as percentage of injected dose per gram (%ID/g). For the tumor model's ex vivo biodistribution, female nude mice bearing BGC823^CLDN18.2^ and BGC823 tumor xenografts were injected by tail vein with 0.74 MBq of [^89^Zr]Zr-DFO-TST001 to evaluate the distribution of [^89^Zr]Zr-DFO-TST001 in major organs and tumors (*n* = 4 per group). The mice were sacrificed and dissected at 48 h p.i. (*n* = 4), and the tumor, kidney, blood, and other major organs were collected and weighed. The blocking study was also performed in BGC823^CLDN18.2^ mice by a co-injection of 0.74 MBq of [^89^Zr]Zr-DFO-TST001 with an excess dose of cold TST001 (1 mg). At 48 h p.i., the blocked mice were sacrificed and dissected. Then, the organ biodistribution of [^89^Zr]Zr-DFO-TST001 was determined.

### 2.5 Dosimetry estimation

For human radiation dosimetry, animal biodistribution data were obtained by the standard method of organ dissection. The human organ radiation dosimetry data were extrapolated from the biodistribution data of [^89^Zr]Zr-DFO-TST001 in KM mice by OLINDA/EXM 2.0 software (Vanderbilt University, Nashville, TN, USA).

### 2.6 Statistical analysis and supplementary experiments

Quantitative data are expressed as the mean ± standard deviation (SD), with all error bars denoting the SD. The means were compared using Student's *t*-test, and *P* values of less than 0.05 were considered as statistical significance. Conjugation and identification DFO-TST001, assessment of CLDN18.2 binding affinity, preparation of [^89^Zr]Zr-DFO-IgG, quality control of [^89^Zr]Zr-DFO-TST001, cell lines and tumor-bearing model, assessment of CLDN18.2 expression, cellular experiments, and immunohistochemistry studies are all described in the Supplementary data.

## 3 Results and discussion

### 3.1 Molecular characteristic of conjugation

**Fig. 1:**
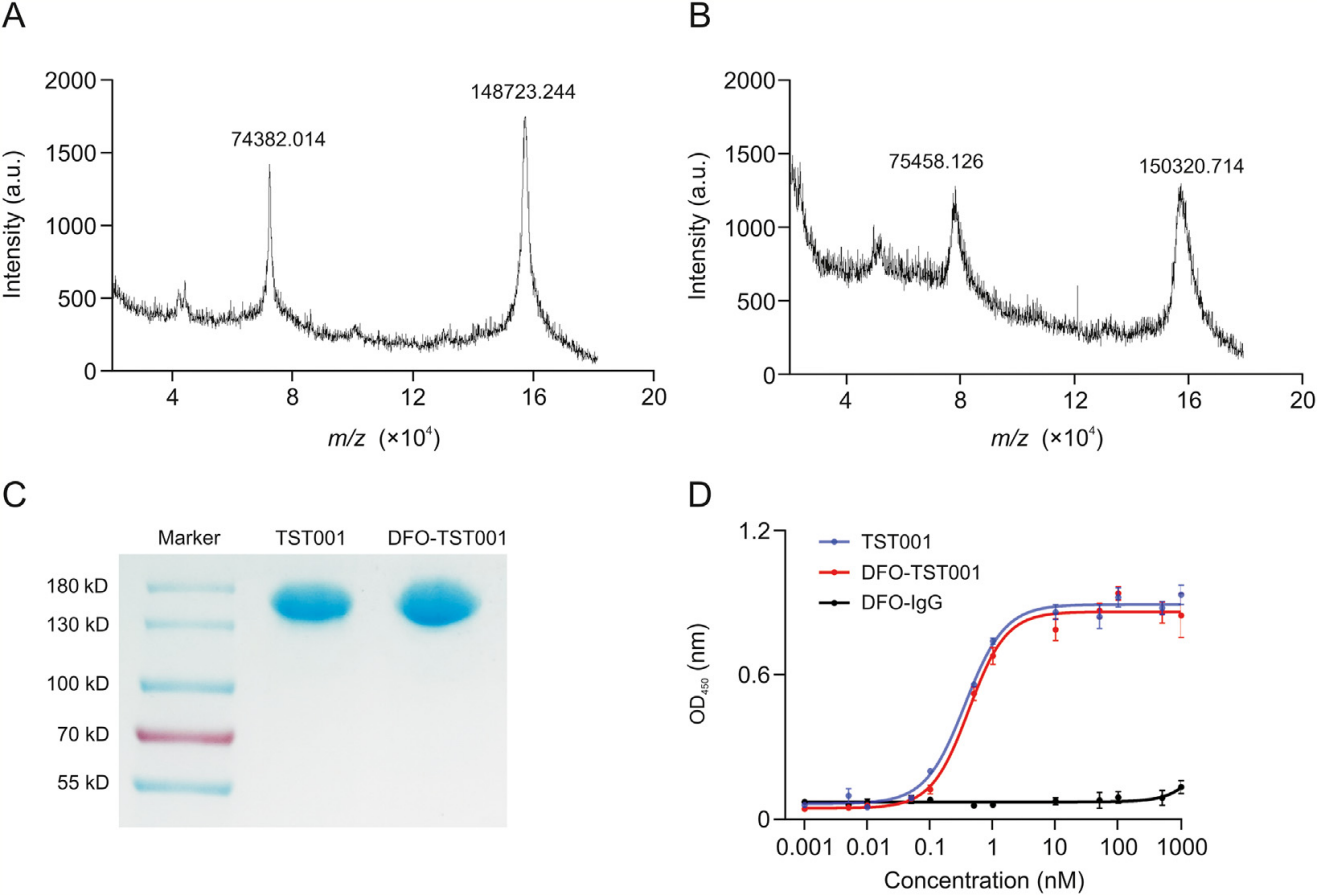
Molecular characterization of TST001 and desferrioxamine (DFO)-TST001. (A) Matrix-assisted laser desorption/ionization time-of-flight mass spectrometry (MALDI-TOF-MS) of TST001. (B) MALDI-TOF-MS of DFO-TST001. (C) Nonreducing sodium dodecyl sulfate-polyacrylamide gel electrophoresis characterization. (D) Binding of TST001 and DFO-TST001 to human CLDN18.2 protein was evaluated by enzyme-linked immunosorbent assays.

The molecular weight of the CLDN18.2 antibody, TST001, was approximately 148 kDa, which was further determined to be exactly 148723.244 Da (Fig. 1A). DFO-TST001 was chelated with an approximately double-DFO chelator with a molecular weight of 150320.714 Da (Fig. 1B). Sodium dodecyl sulfate-polyacrylamide gel electrophoresis showed that both TST001 and DFO-TST001 had bands at approximately 150 kDa with no other bands (Fig. 1C), which indicated that the conjugation was of excellent quality as no antibody aggregates or antibody fragments were detected. The enzyme-linked immunosorbent assays results showed that the EC_50_ value of DFO-TST001 binding to CLDN18.2 was not significantly different from that of TST001 (0.413 ± 0.055 nM vs. 0.361 ± 0.058 nM, *P* > 0.05, Fig. 1D). The binding assay demonstrated both TST001 and DFO-TST001 can form a strong bond with CLDN18.2, and the conjugation of the chelator DFO had no impact on the affinity of TST001 to CLDN18.2.

### 3.2 Radiosynthesis, quality control, and in vitro stability

**Fig. 2:**
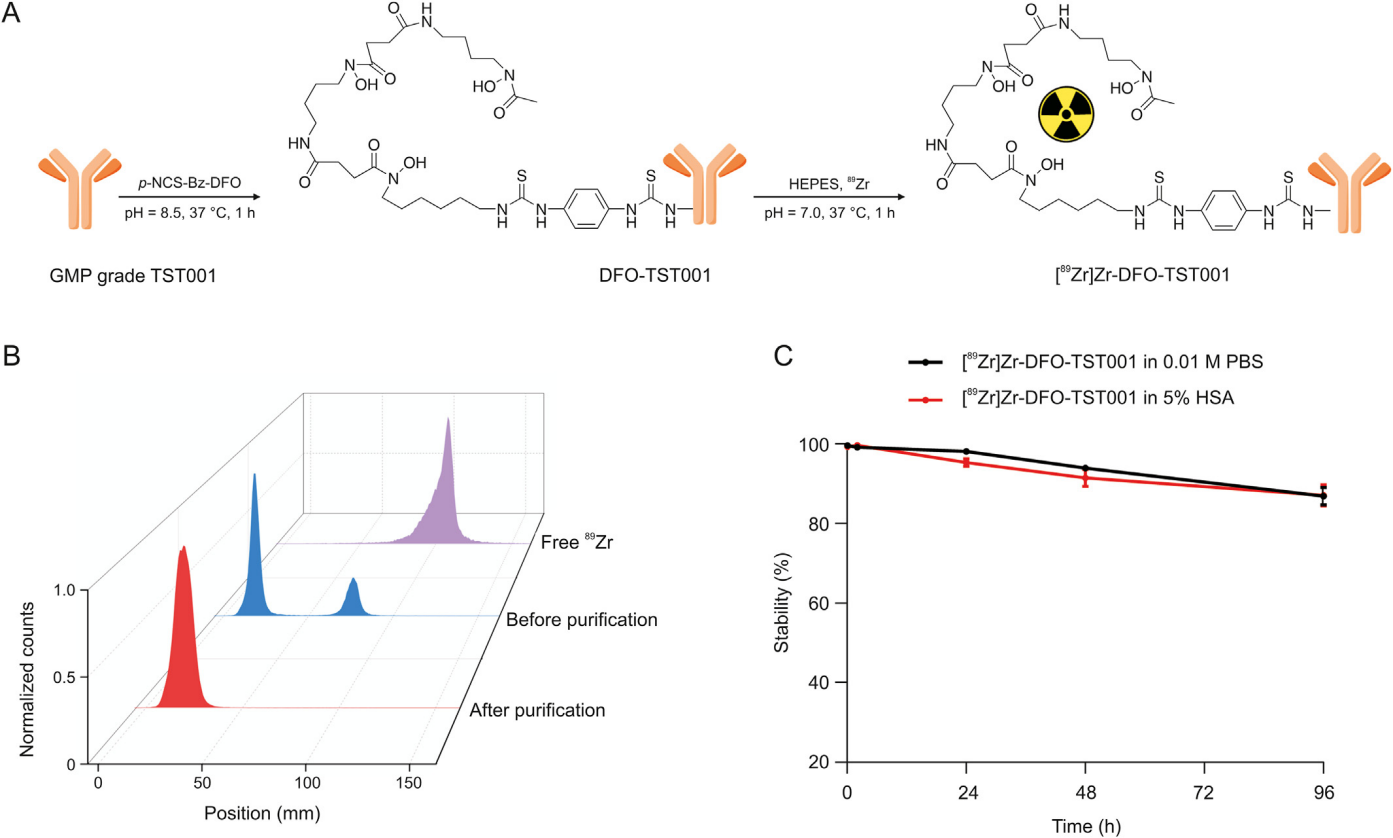
Synthesis, quality control, and vitro stability of [^89^Zr]Zr-desferrioxamine (DFO)-TST001. (A) Synthesis and radiolabeling of [^89^Zr]Zr-DFO-TST001. (B) Radio-thin-layer chromatography scanner results of [^89^Zr]Zr-DFO-TST001 before and after purification. (C) In vitro stability of [^89^Zr]Zr-DFO-TST001. *p*-NCS-Bz-DFO: isothiocyanatobenzyl derivative of DFO; GMP: Good Manufacturing Practice; HEPES: 4-(2-hydroxyethyl)-1-piperazineethanesulfonic acid; PBS: phosphate buffer saline; HSA: human serum albumin.

The synthesis process of [^89^Zr]Zr-DFO-TST001 is shown in Fig. 2A. [^89^Zr]Zr-DFO-TST001 was manually prepared with a radiolabeling yield of 74.64% ± 4.41% (*n* = 3, nondecay corrected). The radiochemical purity (RCP) of [^89^Zr]Zr-DFO-TST001 was more than 99% in 0.01 M PBS (pH 7.4) (Fig. 2B). The in vitro stability of [^89^Zr]Zr-DFO-TST001 in 0.01 M PBS or 5% human serum albumin was demonstrated by an RCP of more than 85% after 96 h at room temperature (Fig. 2C). The excellent in vitro stability also showed that the TST001 structural modification and labeling method was feasible. Quality control results are shown in Table 1.

**Table 1:** Quality control (QC) of [^89^Zr]Zr-DFO-TST001.

Parameter	QC specification	QC result
Appearance	Clear, colorless	Pass
Volume	1–2 mL	1 mL
pH	4.0–8.0	7.4
Radiochemical purity	>95%	>99%
Ethanol	<5%	0
Endotoxins	<15 EU/mL	Pass
Sterility	Sterile	Pass
Specific activity	18.5–296.0 GBq/μmol	24.15 ± 1.34 GBq/μmol

### 3.3 In vitro CLDN18.2 expression of cell lines

**Fig. 3:**
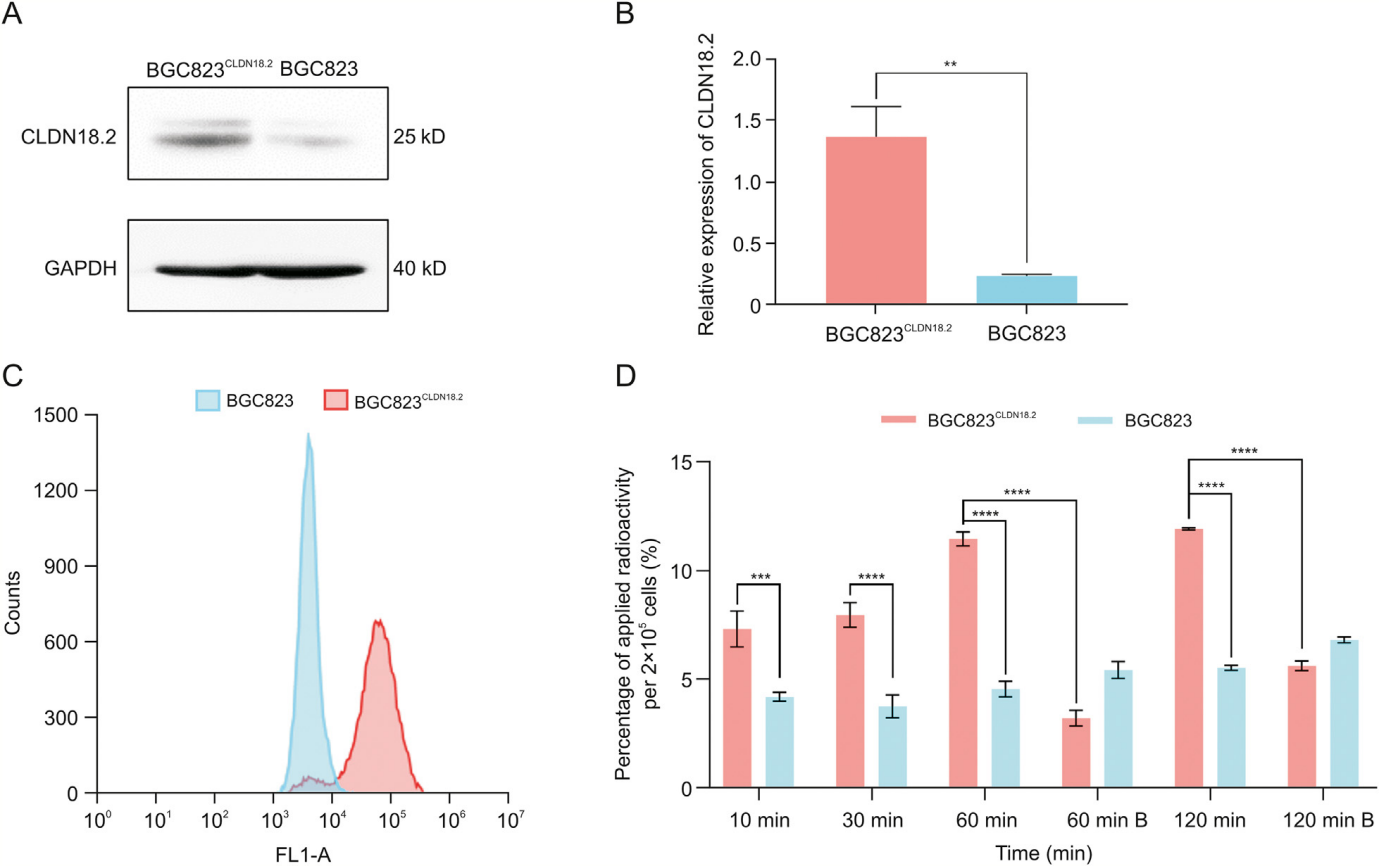
Claudin18.2 (CLDN18.2) expression in two cell lines, and cellular uptake of [^89^Zr]Zr-desferrioxamine (DFO)-TST001. (A) Western blotting results of CLDN18.2 expression in the BGC823^CLDN18.2^ and BGC823 cell lines. (B) Relative expression of CLDN18.2 in BGC823^CLDN18.2^ and BGC823 cells (results are shown as the mean ± standard deviation (SD), *n* = 3). (C) Flow cytometry histogram of BGC823^CLDN18.2^ and BGC823 cells. (D) Cellular uptake of [^89^Zr]Zr-DFO-TST001 in BGC823^CLDN18.2^ and BGC823 cells. B: unlabeled TST001 block. ^∗∗^ *P* < 0.05, ^∗∗∗^*P* < 0.001, ^∗∗∗∗^*P* < 0.0001. GAPDH: glyceraldehyde-3-phosphate dehydrogenase.

Western blotting results confirmed that the expression of CLDN18.2 in BGC823^CLDN18.2^ cells was significantly different from that in BGC823 cells (Fig. 3A). The relative expression of CLDN18.2 in the BGC823^CLDN18.2^ and BGC823 cell lines was 1.37 ± 0.24 and 0.23 ± 0.01, respectively (*P* = 0.0013, Fig. 3B). Flow cytometry experiments revealed that 86.2% of cells were positively stained with anti-CLDN18.2 antibody (1D5) in the BGC823^CLDN18.2^ group (Fig. 3C). The differences in CLDN18.2 expression measured by Western blotting and flow cytometry were then validated between the human gastric cancer cell lines BGC823 and BGC823^CLDN18.2^. The result of the cellular uptake experiment showed that the uptake of [^89^Zr]Zr-DFO-TST001 in BGC823^CLDN18.2^ cells increased in a time-dependent manner (7.33% ± 0.84% at 10 min, 7.97% ± 0.56% at 30 min, 11.47% ± 0.32% at 60 min, and 13.37% ± 2.04% at 120 min), while no significant changes were observed in the BGC823 group (4.21% ± 0.21% at 10 min, 3.77% ± 0.53% at 30 min, 4.57% ± 0.36% at 60 min, and 5.54% ± 0.21% at 120 min). The uptake by BGC823^CLDN18.2^ cells (CLDN18.2 positive) was significantly higher than that by BGC823 cells (CLDN18.2 negative) at each selected time point (*P* < 0.0004). Meanwhile, an excess of unlabeled TST001 significantly blocked the uptake of [^89^Zr]Zr-DFO-TST001 (11.47% ± 0.32% vs. 3.24% ± 0.36% at 60 min, 13.37% ± 2.04% vs. 5.64% ± 0.21% at 120 min) (Fig. 3D). In the cellular uptake experiment, the uptake of [^89^Zr]Zr-DFO-TST001 by BGC823^CLDN18.2^ cells at 60 min was 2.51-fold higher than that of BGC823 cells and 3.54-fold higher than that of the blocking group. The specificity of [^89^Zr]Zr-DFO-TST001 for CLDN18.2 was thus demonstrated at the cellular level.

### 3.4 Dosimetry estimation

The biodistribution study of [^89^Zr]Zr-DFO-TST001 demonstrated favorable pharmacokinetics with a relatively long half-life in vivo (Fig. S1A). Human organ radiation dosimetry is shown in Table 2. The liver received the highest dose (0.360 mSv/MBq), followed by the lungs (0.2000 mSv/MBq). The effective dose was 0.0705 mSv/MBq. When a patient was injected with 74 MBq of [^89^Zr]Zr-DFO-TST001 for imaging, its effective radiation dose was less than 5.217 mSv, which is acceptable in routine nuclear medicine research. The estimated human radiation burden due to a single i.v. [^89^Zr]Zr-DFO-TST001 injection is comparable to that of other ^89^Zr-labeled monoclonal antibodies [[22], [23], [24]], and is suitable for clinical research.

**Table 2:** Estimates of the mean absorbed radiation dose.

Organ	mSv/MBq
Adrenals	0.1310
Brain	0.0308
Oesophagus	0.0794
Eyes	0.0155
Gallbladder wall	0.1550
Left colon	0.0365
Small intestine	0.0586
Stomach wall	0.0615
Right colon	0.0482
Rectum	0.0296
Heart wall	0.0919
Kidneys	0.1330
Liver	0.3600
Lungs	0.2000
Pancreas	0.0666
Prostate	0.0118
Salivary glands	0.0141
Red marrow	0.0461
Osteogenic cells	0.1090
Spleen	0.1380
Testes	0.0048
Thymus	0.0537
Thyroid	0.0368
Urinary bladder wall	0.0081
Total body	0.0272
**Effective dose**	0.0705

### 3.5 Small-animal PET/CT imaging and IHC analysis

Small-animal PET/CT imaging at different time points (2, 24, 48, 72, and 120 h) after injection of [^89^Zr]Zr-DFO-TST001 into KM mice, showed high uptake in the heart, liver, and spleen (Fig. S1B). The SUVmean of some organs measured by ROIs is shown in Fig. S1C. After 2 h, the SUVmean was 2.57 ± 0.02 in the heart, 2.27 ± 0.01 in the liver, and 1.86 ± 0.01 in the spleen, respectively. The ratio of heart to muscle was 20.30 ± 0.91. After 120 h, the SUVmean in the heart, liver and spleen were 0.49 ± 0.01, 1.36 ± 0.02, and 1.21 ± 0.01, respectively, and almost no special intake was observed in the stomach. The images are consistent with the biodistribution results.

**Fig. 4:**
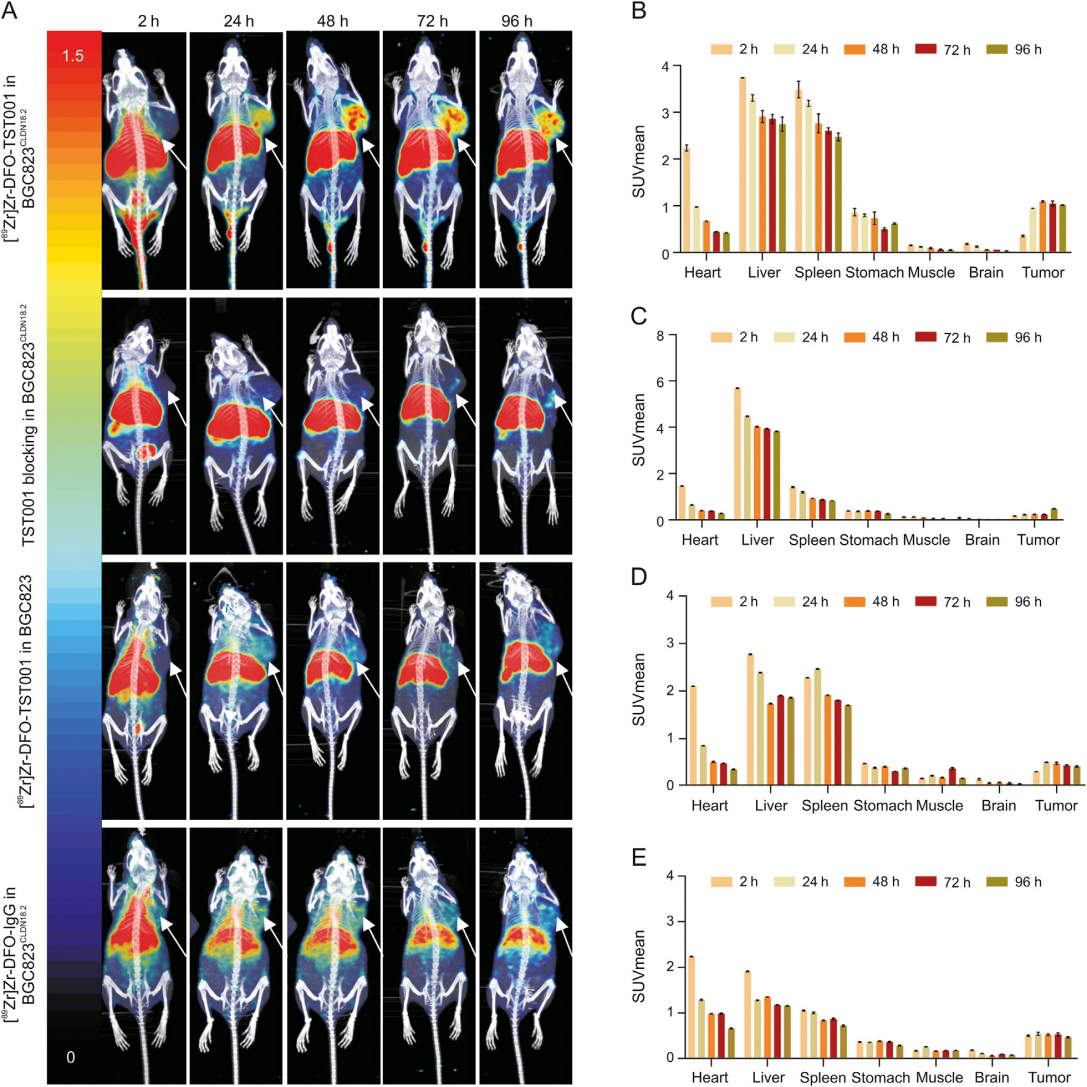
Small-animal positron emission tomography (PET) images of BGC823^CLDN18.2^ or BGC823 tumor mice injected with [^89^Zr]Zr-desferrioxamine (DFO)-TST001 or [^89^Zr]Zr-DFO-IgG. (A) Small-animal PET images of four different groups at 2, 24, 48, 72, and 96 h. Standard uptake value average (SUVmean) of [^89^Zr]Zr-DFO-TST001 in the organs of (B) BGC823^CLDN18.2^ mice, (C) BGC823^CLDN18.2^ mice with unlabeled TST001 blockade, and (D) BGC823 mice. (E) SUVmean of [^89^Zr]Zr-DFO-IgG in organs of BGC823^CLDN18.2^ mice. CLDN18.2: Claudin18.2.

The in vivo distribution and metabolic characteristics of [^89^Zr]Zr-DFO-TST001 were evaluated in real time and noninvasively via small-animal PET/CT imaging at 2, 24, 48, 72, and 96 h p.i. of the radiotracer. Meanwhile, we set up the following three control groups, which were blocked by excess TST001, negative CLDN18.2 expression in BGC823 cells, and nonspecific targeting of [^89^Zr]Zr-DFO-IgG (7.4 MBq), respectively (Fig. 4A). SUVmean data were collected for organs of BGC823^CLDN18.2^ or BGC823 mice by outlining the ROI from the immune-PET images (Figs. 4B−E). The tumor sites in the [^89^Zr]Zr-DFO-TST001 group still had obvious uptake at 96 h p.i.. In the BGC823^CLDN18.2^ model with [^89^Zr]Zr-DFO-TST001, the SUVmean continued to increase within 48 h p.i. and reached a maximum uptake value of 1.09 ± 0.03 at 48 h. In addition, until 96 h p.i., the SUVmean of the BGC823^CLDN18.2^ model was significantly different from that of the BGC823 model and blocking group (1.03 ± 0.03, 0.41 ± 0.05, 0.51 ± 0.07, respectively, *P* < 0.0002) (Fig. S2E). Using [^89^Zr]Zr-DFO-IgG as a negative control probe, the results showed that in the BGC823^CLDN18.2^ model mice except for the tumor uptake value slightly higher than [^89^Zr]Zr-DFO-TST001 at 2 h after injection (0.51 ± 0.01 vs. 0.37 ± 0.02), the [^89^Zr]Zr-DFO-IgG tumor uptake value at all other time points (24, 48, 72, and 96 h) was significantly lower than that of [^89^Zr]Zr-DFO-TST001 (0.55 ± 0.04 vs. 0.96 ± 0.12, 0.53 ± 0.02 vs. 1.10 ± 0.12, 0.54 ± 0.04 vs. 1.06 ± 0.06 and 0.47 ± 0.01 vs. 1.03 ± 0.01) (Fig. S2). Over time, compared with other imaging groups, the uptake of [^89^Zr]Zr-DFO-TST001 was mostly concentrated in the tumor in the BGC823^CLDN18.2^ model, and the uptake values of the heart, liver, and other organs were greatly reduced (Fig. 4A).

**Fig. 5:**
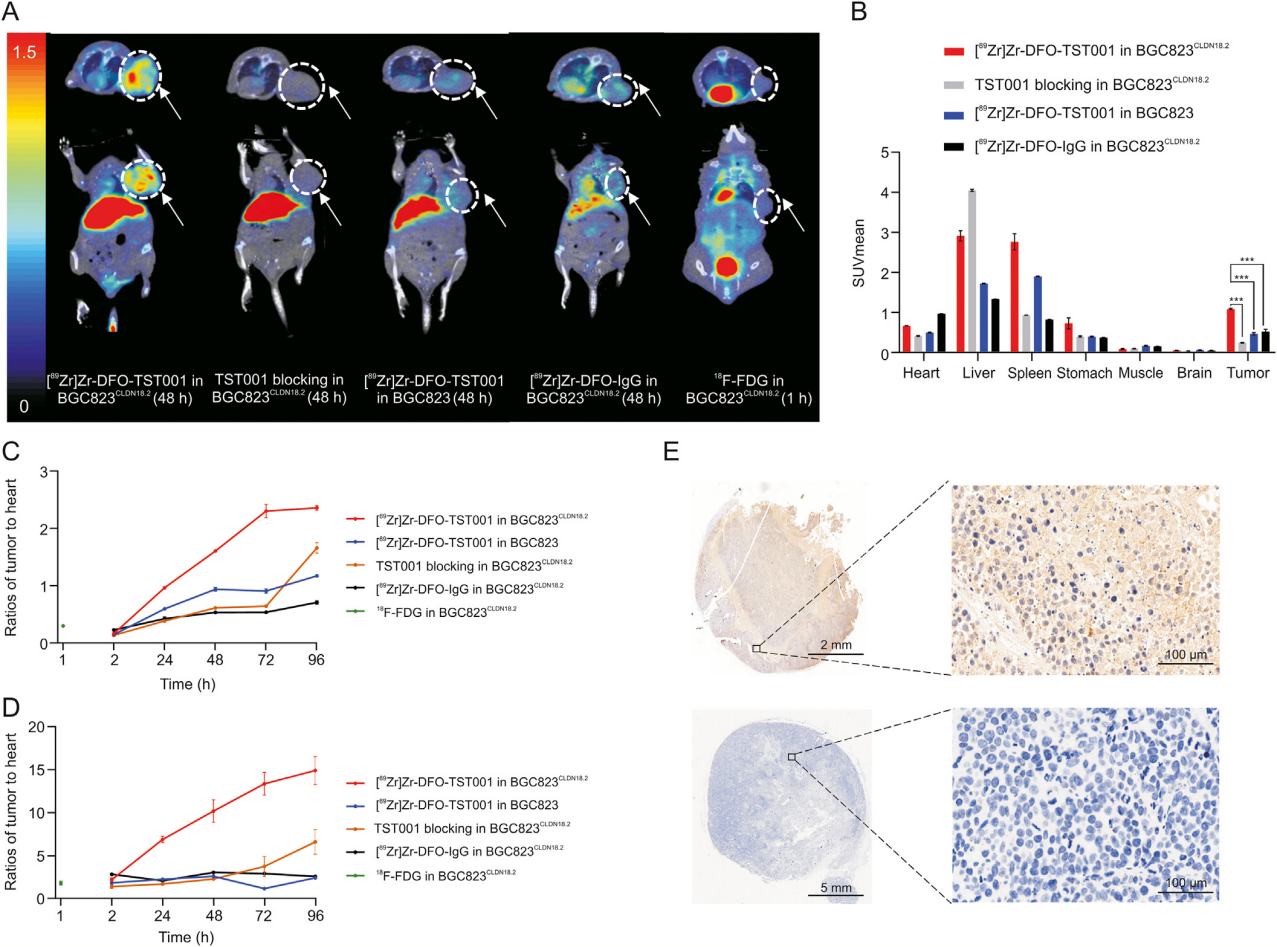
Analysis of small-animal positron emission tomography (PET) imaging. (A) Section images of tumor uptake 48 h p.i. were compared to section images of ^18^F-fluorodeoxyglucose (^18^F-FDG) in BGC823^CLDN18.2^ mice 1 h p.i. (B) Standard uptake value average (SUVmean) in the organs of different experimental group mice in organs at 48 h. (C) Tumor/heart at each point p.i.. (D) Tumor/muscle at each point p.i.. (E) Immunohistochemistry (IHC) analysis of CLDN18.2 expression in BGC823^CLDN18.2^ (++) (top) and BGC823 (−) (bottom) tumors. ^∗∗∗^*P* < 0.001. DFO: desferrioxamine; CLDN18.2: Claudin18.2.

For comparison with the gold-standard probe ^18^F-FDG, BGC823^CLDN18.2^ tumor-bearing mice were given ^18^F-FDG and images were collected 1 h p.i. (Fig. 5A). The results showed that the uptake of ^18^F-FDG in CLDN18.2-positive mice was similar to the background uptake. The tumor accumulation of [^89^Zr]Zr-DFO-TST001 in BGC823^CLDN18.2^ mice 48 h p.i. was approximately 4.11-fold that of the blocking group, 2.27-fold that of the BGC823 group, and 2.06-fold that of the [^89^Zr]Zr-DFO-IgG group (SUVmean values were 1.11 ± 0.02, 0.27 ± 0.01, 0.49 ± 0.03, 0.54 ± 0.06, respectively) (Fig. 5B). The tumor/heart (T/H) ratios and tumor/muscle (T/M) ratios at each time point after injection of [^89^Zr]Zr-DFO-TST001 were significantly higher than those of the other control groups (Figs. 5C and D), and at 96 h p.i., the T/H and T/M ratios reached their maximum of 2.37 ± 0.04, 14.95 ± 1.63, respectively.

Compared with our previous research, TST001 is a humanized antibody with better immune responsiveness to the CLDN18.2 receptor. Second, the patient needs to receive iodine to block the thyroid gland before and during ^124^I imaging, which greatly reduces patient compliance [21]. Labeling with ^89^Zr would appear to be more robust and better available. Nevertheless, a remarkably high background in the liver and spleen was also noted with [^89^Zr]Zr-DFO-TST001, which might be a result of nonspecific binding and hepatobiliary clearance. This is very similar to previous studies on the ^89^Zr-labeled antibody [25,26]. From an imaging perspective, this not only results in problems for tumor localization in the liver and spleen region, but it also might lead to false-positive results when “tumor CLDN18.2 expression” and further cause erroneous selection of candidate patients for this therapy. Although the interactions between FcγR expressed on immune effector cells and the Fc region of antibodies can trigger antibody-mediated therapeutic responses, they may not be favorable in the context of molecular imaging. There are three initial resolutions to reduce nonspecific uptake by the liver and spleen [27,28]. Firstly, the preparation of probes using antibody fragments such as Fab and F(ab)_2_ to replace intact antibodies not only avoids the interaction of the Fc region with the immune system, but also allows the probes to have a faster pharmacokinetic profile. Secondly, another strategy is predicated on genetically engineering the Fc region of an IgG to abrogate its binding with FcγRs on immune cells while maintaining its ability to bind FcRn. Thirdly, a more facile and modular approach may lie in manipulating the glycans of the Fc region. In addition, from the nature of the nuclide, ^89^Zr is a radioactive metal ion that first ligates the antibody by a suitable chelating agent (typically using a lysine group) and then indirectly labels the antibody by non-covalently chelating the radioactive metal ion. Once antibodies have been internalized into the tumor cells, they are subject to catabolism through lysosomal degradation. The catabolites of radiometal ion chelates remain trapped (residualized) inside the cells, leading to an accumulation of radiometal (and PET signal) in the target tumor tissue and metabolic organ over time. However, iodine is usually labeled directly onto antibodies through a simple and widely used procedure, and most iodine-containing catabolites are nonpolar molecules that are rapidly lost from the liver and spleen [29]. Based on this property of radionuclide iodine, we are also conducting a study related to ^124^I labeled TST001, which may be more suitable for clinical translation in the future.

We also performed ^18^F-FDG PET/CT imaging as a reference. The tumor uptake of [^89^Zr]Zr-DFO-TST001 was higher than that of ^18^F-FDG at the tumor sites in the BGC823^CLDN18.2^ model, and the T/M value of [^89^Zr]Zr-DFO-TST001 was also much higher than that of ^18^F-FDG (10.23 ± 1.30 vs. 1.80 ± 0.22) (Figs. 5A and D).

The results of IHC revealed high and homogenous CLDN18.2 expression in BGC823^CLDN18.2^ tumors, and the BGC823 xenograft tumors were negative for CLDN18.2 (Fig. 5E). The stomachs of BGC823^CLDN18.2^ and BGC823 tumor-bearing mice showed substantially positive expression of CLDN18.2. Neither the liver nor spleen tissue of the two types of tumor-bearing mice expressed CLDN18.2. The IHC results showed that the BGC823^CLDN18.2^ tumors were strongly positive for CLDN18.2 (+++), while the BGC823 tumors were negative (−), which was consistent with the imaging and Western blotting results (Fig. S3). These results prove that the [^89^Zr]Zr-DFO-TST001 probe we constructed has the ability to specifically target CLDN18.2. In addition, a strong positive expression of CLDN18.2 (+++) was also observed in the gastric mucosa of all mice, but neither PET/CT imaging nor biodistribution showed any obvious uptake and retention of the probe in the stomach, likely because the expression of CLDN18.2 in vivo was limited to the gastric mucosa, and monoclonal antibodies had difficulty accessing the hidden CLDN18.2 binding epitope in the gastric mucosa [30].

### 3.6 Ex vivo biodistribution

**Fig. 6:**
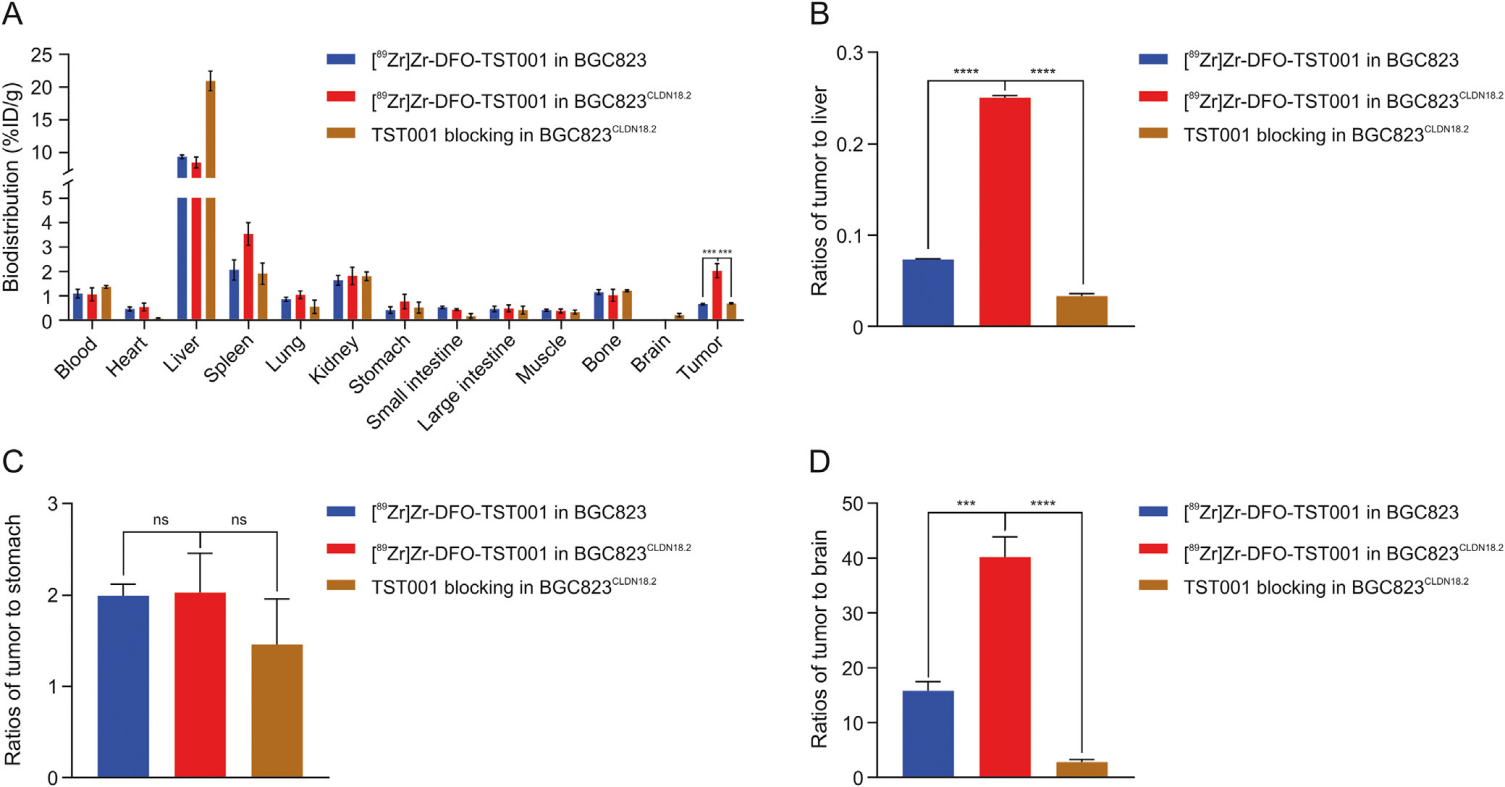
Biodistribution studies of radio-tracer in each tumor model. (A) Biodistrbution of [^89^Zr]Zr-DFO-TST001 in tumor models 48 h p.i.. (B) Tumor/Liver 48 h p.i.. (C) Tumor/Stomach 48 h p.i.. (D) Tumor/Brain 48 h p.i.. ^∗∗∗^*P* < 0.001; ^∗∗∗∗^*P* < 0.0001; ns: no significant difference in statistics. %ID/g: percentage of injected dose per gram; DFO: desferrioxamine; CLDN18.2: Claudin18.2.

The biodistribution of [^89^Zr]Zr-DFO-TST001 in BGC823^CLDN18.2^ and BGC823 tumor-bearing mice is presented in Fig. 6. At 48 h p.i., the livers in all three groups showed relatively high uptake (8.39 ± 0.59 %ID/g in BGC823^CLDN18.2^ group, 9.28 ± 0.19 %ID/g in BGC823 group, and 20.96 ± 0.88 %ID/g in blocking group, respectively). The uptake value of the spleen was second to that of the liver (3.54 ± 0.26 %ID/g in BGC823^CLDN18.2^ group, 2.08 ± 0.29 %ID/g in BGC823 group, and 1.93 ± 0.24 %ID/g in blocking group, respectively). Tumor uptake in BGC823^CLDN18.2^ tumor bearing mice was higher (2.05 ± 0.16 %ID/g) than that in the BGC823 mice (0.69 ± 0.02 %ID/g) and blocking group (0.72 ± 0.02 %ID/g) (Fig. 6A). The tumor/liver (T/L) and tumor/brain (T/B) ratios of BGC823^CLDN18.2^ tumors were significantly higher than those of the other two control groups (T/L: 0.075 ± 0.001 in the BGC823 group vs. 0.25 ± 0.003 in the BGC823^CLDN18.2^ group vs. 0.035 ± 0.002 in the blocking group; T/B: 16.03 ± 1.66 in the BGC823 group vs. 40.35 ± 3.68 in the BGC823^CLDN18.2^ group vs. 3.01 ± 0.53 in the blocking group) (Figs. 6B and D). The tumor/stomach ratios were not significantly different among the three groups (2.00 ± 0.13 in BGC823 vs. 2.04 ± 0.43 in BGC823^CLDN18.2^ vs. 1.47 ± 0.50 in blocking group) (Fig. 6C). Consistent with the PET/CT results, in vitro biodistribution data at 48 h p.i. showed that [^89^Zr]Zr-DFO-TST001 aggregated in the liver and spleen, and the liver uptake in the blocking group was significantly higher than that in the other two groups, possibly because tumor uptake was blocked, resulting in the probes entering the liver directly through the bloodstream for metabolism. The difference in tumor uptake values in the three groups also reflects the excellent specificity of [^89^Zr]Zr-DFO-TST001 for CLDN18.2-positive tumors.

## 4 Conclusion

We successfully prepared ^89^Zr labeling of a GMP grade anti-CLDN18.2 recombinant humanized antibody TST001. [^89^Zr]Zr-DFO-TST001 exhibited good specificity at the cellular level and rapid tumor accumulation which remained positive from 24 to 96 h. It provides a promising molecular probe for detecting the treatment effects of therapeutic antibodies in humans in real time. It also provides a possibility for the screening and efficacy evaluation of patients targeted for CLDN18.2 therapy in the future.

## CRediT author statement

**Yan Chen** and **Xingguo Hou:** Investigation, Methodology, Software, Formal analysis, Data curation, Writing - Original draft preparation; **Dapeng Li:** Investigation, Methodology, Software, Writing - Original draft preparation; **Jin Ding:** Conceptualization, Investigation, Resources, Validation; **Jiayue Liu:** Methodology, Software, Formal analysis; **Zilei Wang:** Methodology, Software, Formal analysis; **Fei Teng**, **Hongjun Li**, **Fan Zhang**, **Yi Gu**, and **Steven Yu:** Resources, Validation, Supervision; **Xueming Qian:** Investigation, Resources, Validation, Supervision; **Zhi Yang:** Conceptualization, Methodology, Investigation, Resources, Validation, Supervision; **Hua Zhu:** Conceptualization, Methodology, Investigation, Resources, Validation, Writing - Reviewing and Editing, Supervision.

## Declaration of competing interest

Intellectual properties protection have been filed by Suzhou Transcenta Therapeutics Co., Ltd., inventor of Xueming Qian; Fei Teng; Hongjun Li; Yi Gu, and Beijing Cancer Hospital, inventor of Hua Zhu; Yang Zhi; Jin Ding; Feng Wang. All authors declare that they have no known competing financial interests or personal relationships that could have appeared to influence the work reported in this paper.
