# Supplementary material for: Development of a CLDN18.2-targeting immuno-PET probe for non-invasive imaging in gastrointestinal tumors
Source: J Pharm Anal. 2023 Feb 28;13(4):367–75. doi: 10.1016/j.jpha.2023.02.011 (PMC10173170; doi:10.1016/j.jpha.2023.02.011)
Supplement: Multimedia component 1 [file mmc1.docx]

# *Supporting material*

**Development of a CLDN18.2-targeting Immuno-PET Probe for Non-invasive Imaging in Gastrointestinal Tumors**

## Supplementary materials and methods

Conjugation and identification DFO-TST001

Both modification and radiolabeling methods for TST001 have been reported in previous studies. Specifically, TST001 (3.3 nmol) were dissolved in NaHCO_3_ buffer (0.1 M, pH 9.5) after exchanging the solvent with 0.01M PBS. The pH value was adjusted to 9.0 with 0.1 M Na_2_CO_3_ solution. 60 nmol p-NCS-Bz-DFO dissolved in DMSO was then added to the above solution with a molar ratio of p-NCS-Bz-DFO to TST001 as 20:1. After mixing and reacting at 37 °C for 1 h, the crude product was further purified by PD-10 column (2.5 ml, 0.01 M PBS) and stored at -80 °C in stabilizer.

The mass spectra of TST001 and DFO-TST001 were measured by matrix-assisted laser desorption/ionization time-of-flight mass spectrometry (MALDI-TOF-MS) (Bruker Dalton, Germany). For nonreducing sodium dodecyl sulfate-polyacrylamide gel electrophoresis (SDS-PAGE), 10 μg antibody samples were diluted with 0.01 M PBS and ×5 nonreducing sample buffer without dithiothreitol, then separated on an 6% sodium dodecyl sulfate PAGE gel by electrophoresis. The gel was stained with 0.5% Coomassie blue.

Assessment of CLDN18.2 binding affinity

Enzyme-linked immunosorbent assays (ELISAs) were used to determine the binding potency between TST001 and DFO-TST001 with human CLDN18.2 full length protein-VLP (CL2-H52P7) (ACRO Biosystems, Beijing, China). First, 100 μL solution of the CL2-H52P7 (2 μg/mL) was added to each well coated with a 96-well polystyrene StripwellTM microplate (Corning Costar, CLS2481-100EA), at 4 °C overnight. Then, the antigen solution was discarded, and the protein was washed five times with PBST (0.01 M pH 7.4 PBS and 0.2% Tween-20). After that, 5% powdered milk (diluted with PBS) was added to the microplate for 2 h at 37 ℃ to block other nonspecific sites. Then, after discarding the 5% powdered milk, five times washed with PBST. After the plate was washed, the diluted sample (IgG, TST001 and DFO-TST001) was added with seven concentration gradients, 0.001, 0.005, 0.01, 0.05, 0.1, 0.5, 1,10,50, 100, 500 and 1000 nM to the microplate (100 μL/well) and the membrane was covered for 2 h at 15-25 °C. The plate was washed again, and secondary antibody was added: goat anti-mouse IgG4 Fc (HRP) (Abcam, shanghai, China) was diluted 1:3000 in the enzyme-labeled plate with a secondary antibody diluent (100 μL/well) and incubated at 15-25 °C for 1 h. For color development after washing, coloring solution (100 μL/well) was added, and the plate was covered with film and developed while protected from light at 15-25 °C for 20 min. For termination of the reaction, stop solution was added (50 μL/well. For detection, the optical density (OD) value of each well was read by a microplate reader (BIO-RAD, CA, USA) at a detection wavelength of 450 nm. The 50% maximal effect (EC_50_) value was also used to assess the affinity of anti-CLDN18.2 antibodies to CLDN18.2.

**Preparation of [^89^Zr]Zr-DFO- IgG**

The preparation of DFO-IgG is the same as that of DFO-TST001. For ^89^Zr labeling, ^89^Zr-oxalic acid was neutralized to pH 7.0 using 0.25 M 2-[4-(2-hydroxyethy)-1-piperazinyl］ethanesulfonic acid (HEPES) and 1 M Na_2_CO_3_ buffer , then mixed with DFO-IgG for 60 min 37 °C. The reaction mixture was purified by PD-10 column (2.5 ml, 0.01 M PBS).

Quality control of [^89^Zr]Zr-DFO-TST001

Quality control of [^89^Zr]Zr-DFO-TST001 tracer is carried out in accordance with the Guidelines for Quality Control of Positron Radiopharmaceuticals.

1. pH: Take 1 drop of radiopharmaceuticals, drop it on a precision pH test strip, and compare it with the standard color swatch, that is, the pH value of the solution.
2. Ethanol content: No ethanol is added during the preparation of the tracer preparation, and there is no need for inspection.
3. Endotoxin: Take this product, according to the Chinese Pharmacopoeia (2020) General Rule 1143 for inspection, this product should contain less than 15 EU/mL of endotoxin.
4. Sterility: Take this product, according to the Chinese Pharmacopoeia (2020) General Rule 1101 for inspection.
5. Specific activity: Refers to the activity of an element of a radionuclide or the unit mass of its compound.

Radiochemical purity and vitro stability

The radiochemical yield and radiochemical purity were measured by Radio-thin-layer chromatography scanner (Radio-TLC) (Bioscan, Santa Barbara, CA, USA) in a standard protocol. The pre-purification and post-purification products [^89^Zr]Zr-DFO-TST001 (2uL) were dropped to the lower end of the TLC-SG test strip 1 cm, and then the TLC-SG test strip was placed in the eluent 0.5 M sodium citrate (PH=5.0) buffer. When the eluent is unfolded to 10 cm from the lower end, it is removed and dried, radio-TLC analysis is performed, and the Rf value is calculated. The Rf values of free Zr-89 and [^89^Zr]Zr-DFO-TST001 are 0.9-1.0 and 0-0.1 respectively. The vitro stability study is conducted that [^89^Zr]Zr-DFO-TST001 incubated with 0.01 M phosphate buffer saline (PBS) or 5% (human serum albumin) HSA at room temperature (RT). Radio-TLC were performed at various incubation time (0, 2, 12, 24, 48, and 96 h).

Cell lines and tumor-bearing model

The human stomach cancer cell line BGC823 was obtained at Peking University Cancer Hospital and Institute (Beijing, China). The BGC823^CLDN18.2^ cell line was generated by transfection with the full-length CLDN18.2. The cells were cultured in RPMI-1640 medium which was supplemented with 10% FBS plus antibiotics from Invitrogen. All animal experiments were performed according to the National Institutes of Health guidelines for the care and use of laboratory animals and approved by the Animal Care and Ethics Committee of Peking University Cancer Hospital. For PET/CT imaging and vitro biodistribution experiments, BGC823 and BGC823^CLDN18.2^ xenografts were established in 4 to 6-week-old female BALB/c nu/nu mice which were purchased from Beijing Vital River Laboratory Animal Technology Co., Ltd (Beijing, China). The right axillary of the mouse was subcutaneously injected with 1 × 10^6^ BGC823/BGC823^CLDN18.2^ cells suspended in 100 μL PBS. Tumors were grown for 3 weeks to reach an average volume of 100 mm^3^.

Assessment of CLDN18.2 expression

Western blotting was performed as previously described. After washing three times with cold PBS, 500 μL of RIPA lysis buffer was added to each cell dish for 10 min in an ice box. The cell lysate was centrifuged for 5 min at 15000 rpm at 4 °C, and the supernatant was collected and stored on ice. Equal amounts of supernatant (20 μg) were separated to extract protein by SDS−PAGE (250 mA, 90 min), and transferred over to a polyvinylidene fluoride (PVDF) membrane (Merck Millipore, Boston, MA, USA). Blots were incubated with the following primary antibodies: rabbit anti-human CLDN18.2 (ab213480, 1:500) (Abcam, Shanghai, China). Blots were then incubated with an HRP-conjugated goat anti-rabbit secondary antibody (1:10000) (Abcam, Shanghai, China). Proteins were detected using to Clarity Western ECL Substrate (YEASEN Biotech, Shanghai, China). Immunoblots were imaged with the Alliance Micro Q9 chemiluminescence imaging system (Alliance Micro Q9, UVITEC, Britain). Cells (2 × 10^5^) were collected and washed with cold PBS twice, stained with 2 μg/mL CLDN18.2 antibody (1D5, Beijing cancer hospital, Beijing, China) for 1 h at room temperature followed by Alexa Fluor 488-conjugated antibody for 30 min, and then subjected to flow cytometry with BD FACS Aria Flow cytometric analyses were performed on a Beckman Coulter Cytomics FC 500 MPL (BD, NJ, USA).

Cellular experiments.

BGC823/BGC823^CLDN18.2^ cells were cultured in RPMI-1640 culture medium (2.0 × 10^5^ cells/mL) and added to a 24-well plate (1.0 mL per well) to culture overnight. [^89^Zr]Zr-DFO-TST001 (20 μL, 37 kBq, 6.87 × 10^−12^ M) was added to wells (n = 4) containing adherent BGC823/BGC823^CLDN18.2^ cells. The mixture was incubated in a 5% CO_2_ incubator at 37 °C for 2, 10, 30, 60, and 120 min. After incubation, the culture medium was removed and the cells were washed 2 times with cold PBS (0.01 M). Inhibition for 60 and 120 min was performed in the presence of excess unlabeled TST001 (50 μg). Then, the cells were collected after digestion by 1 M NaOH and counted in a γ counter. The percentage of added dose per 2.0 × 10^5^ cells (%AD/2.0 × 10^5^ cells) was calculated according to the count.

Immunohistochemistry studies

Paraffin sections were deparaffinized with xylene. Following rehydration in distilled water, antigen was retrieved by heating in EDTA (pH 9.0) for 10 min. Endogenous peroxidase activity was blocked by incubating in 3% hydrogen peroxide at room temperature for 15 min. Nonspecific binding was blocked with goat blocking serum for 1 h at room temperature. Anti-CLDN18.2 rabbit monoclonal antibody (Abcam, ab222512, Shanghai, China) diluted at 1:500 was added, and the slides were incubated at 4 °C overnight. Following three washes, the slides were incubated with Envision (DAKO) for 45 min at room temperature. Diaminobenzidine was used as a chromogen. Sections were counterstained with hematoxylin, dehydrated, and mounted. Evaluation of immunohistochemical slides was performed using a Leica AT2 microscope (Wetzlar, Germany).

## Figures


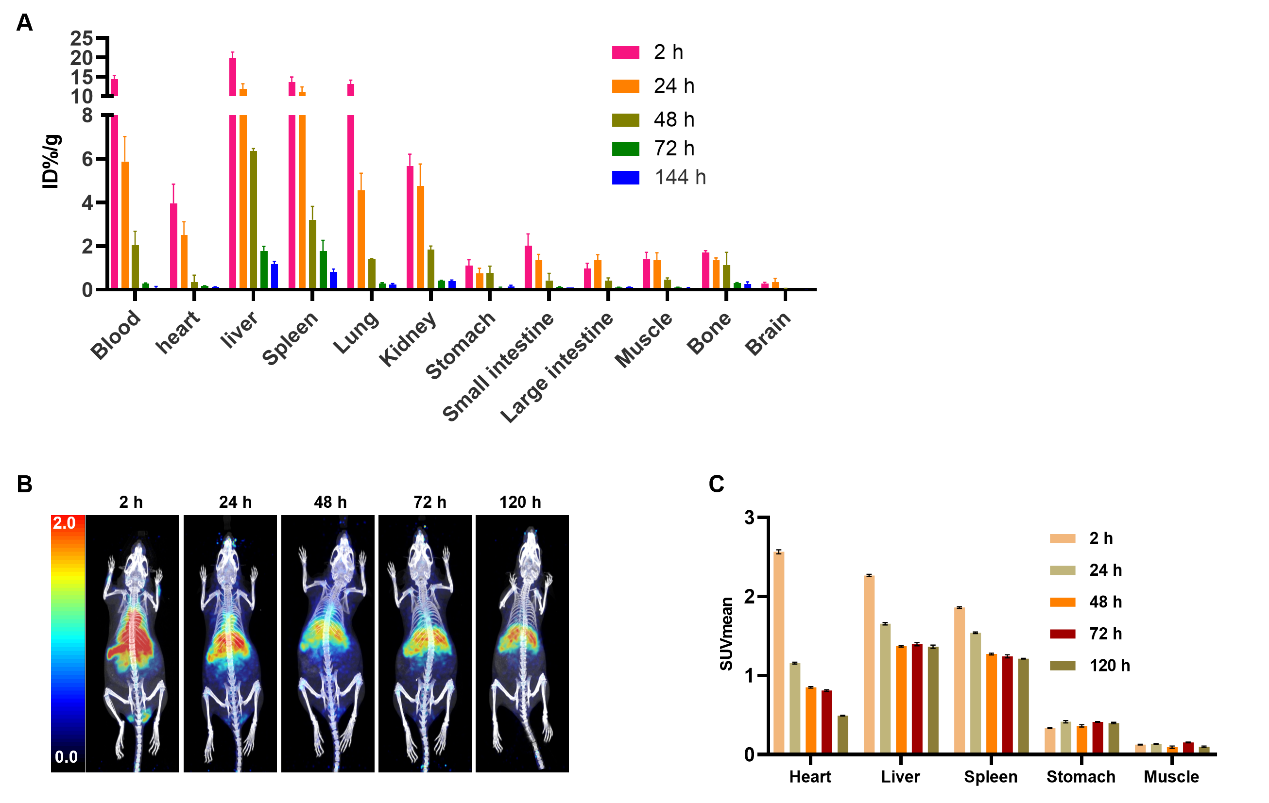


Fig. S1. Biodistribution and small-animal positron emission tomography (PET) imaging of [^89^Zr]Zr-DFO-TST001 in KM mice. (A) The biodistribution of[^89^Zr]Zr-DFO-TST001 in normal mice. (B) Micro-PET/CT imaging of [^89^Zr]Zr-DFO-TST001 in normal mice at 2, 24, 48 ,72 h and 120 h after tail vein injection. (C). Standard uptake value average (SUVmean) of vital organs at different time points.


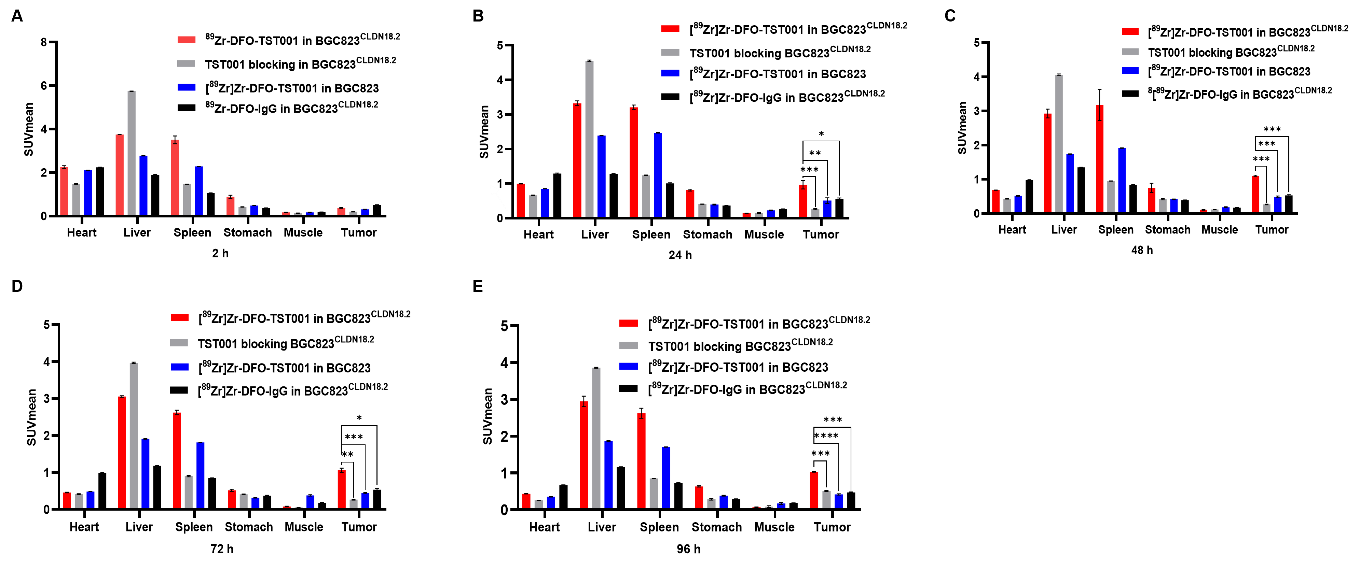


Fig. S2. SUVmean of different experimental group mice in organs at different time points. (A) SUVmean of different experimental group mice in organs at 2 h. (B) SUVmean of different experimental group mice in organs at 24 h. (C) SUVmean of different experimental group mice in organs at 48 h. (D) SUVmean of different experimental group mice in organs at 72 h. (E) SUVmean of different experimental group mice in organs at 96 h. (*, *P* < 0.05; **, *P* < 0.01; ***, *P*< 0.001; ****, *P*< 0.0001).


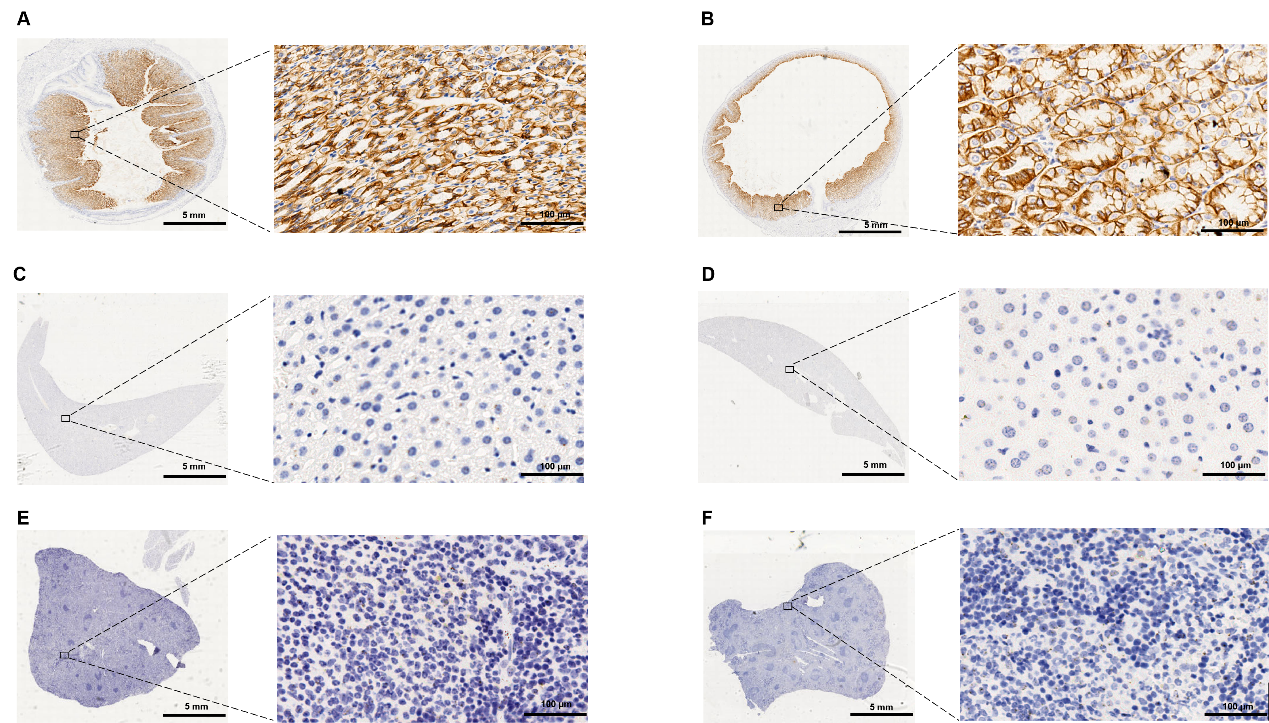


Fig. S3. Immunohistochemistry results of the major organs in BGC823^CLDN18.2^ and BGC823 mice. (A) Stomach in BGC823^CLDN18.2^ model mice (+++). (B) Stomach in BGC823 model mice (+++). (C) Spleen in BGC823^CLDN18.2^ model mice (-). (D) Spleen in BGC823 model mice (-). (E) Liver in BGC823^CLDN18.2^ model mice (-). (F) Liver in BGC823 model mice (-).
